# Severe Embryotoxicity of Artemisinin Derivatives in Experimental Animals, but Possibly Safe in Pregnant Women

**DOI:** 10.3390/molecules15010040

**Published:** 2009-12-25

**Authors:** Qigui Li, Peter J. Weina

**Affiliations:** Division of Experimental Therapeutics, Walter Reed Army Institute of Research, 503 Robert Grant Avenue, Silver Spring, MD 20307–5100, USA

**Keywords:** Artemisinins, artesunate, dihydroartemisinin, embryotoxicity, pharmacokinetics, pregnant animals, pregnant women

## Abstract

Preclinical studies in rodents have demonstrated that artemisinins, especially injectable artesunate, can induce fetal death and congenital malformations at a low dose range. The embryotoxicity can be induced in those animals only within a narrow window in early embryogenesis. Evidence was presented that the mechanism by which embryotoxicity of artemisinins occurs seems to be limited to fetal erythropoiesis and vasculogenesis/ angiogenesis on the very earliest developing red blood cells, causing severe anemia in the embryos with higher drug peak concentrations. However, this embryotoxicity has not been convincingly observed in clinical trials from 1,837 pregnant women, including 176 patients in the first trimester exposed to an artemisinin agent or artemisinin-based combination therapy (ACT) from 1989 to 2009. In the rodent, the sensitive early red cells are produced synchronously over one day with single or multiple exposures to the drug can result in a high proportion of cell deaths. In contrast, primates required a longer period of treatment of 12 days to induce such embryonic loss. In humans only limited information is available about this stage of red cell development; however, it is known to take place over a longer time period, and it may well be that a limited period of treatment of 2 to 3 days for malaria would not produce serious toxic effects. In addition, current oral intake, the most commonly used route of administration in pregnant women with an ACT, results in lower peak concentration and shorter exposure time of artemisinins that demonstrated that such a concentration–course profile is unlikely to induce the embryotoxicity. When relating the animal and human toxicity of artemisinins, the different drug sensitive period and pharmacokinetic profiles as reviewed in the present report may provide a great margin of safety in the pregnant women.

## Introduction

Artemisinin–based combination therapies (ACTs) and injectable artesunate (AS) are currently recommended as the frontline antimalarial treatments for uncomplicated and severe malaria, respectively, with over 100 million courses administered annually [1]. Despite possessing excellent therapeutic activity and tolerability, neurotoxicity and embryotoxicity have been reported in cross–species animal models. Generally, studies in animals are very valuable in indicating possible risks in human from medicines. However, artemisinin and its derivatives are considered safe and effective in pregnant women who have been treated with artemisinin compounds, including a small number in the first trimester. In clinical trials, the patients did not show any increases in miscarriage or stillbirth with abnormality evidence. A follow–up of exposed babies did not reveal developmental delays [2,3].

Dellicour *et al*. reviewed the possible relationship between artemisinin compounds and adverse pregnancy outcomes recently. These authors concluded that current data are limited and the published studies do not have adequate power to rule out rare serious adverse events, even in second and third trimesters. Therefore, there is insufficient evidence to effectively assess the risk–benefit profile of artemisinin compounds for pregnant women, particularly, during first trimester exposure [4]. Comprehensive knowledge of the mechanism(s) involved in embryotoxicity in animals was recognized to be of value in extrapolating the risks for humans. An informal non–clinical consultation meeting was convened in January 2006 to review the findings of animal studies (rodents and primates) performed since 2002 and consider their impact on the clinical use of artemisinins [5]. Current WHO Guidelines was followed by an informal clinical consultation in October 2006 to recommend that “In uncomplicated malaria, ACT treatment should be used in the second and third trimester, but should be used in the first trimester only if it is the only effective treatment available. In severe malaria, artemisinins are preferred over quinine in the second and third trimester because of the hypoglycaemia associated with quinine. However, in the first trimester until more evidence becomes available on the risk benefit ratio of artemisinins, both artesunate and quinine may be considered as options.” [1].

This new recommendation was founded on the recent publications in 2006 [5]. Preclinical studies in rodents have demonstrated that artemisinins can induce fetal death and malformations at high oral dose and low injectable dose levels [4]. The death and malformations can be induced in rodents only within a narrow window in early embryogenesis. Confirmation was presented that the mechanism by which embryotoxicity of artemisinins was produced was through antiangiogenic and antierythropoietic actions on the embryonic erythroblasts in very earliest developing red blood cells causing severe anaemia in the embryo, which studies were drew inspiration from anticancer studies of artesunate [6,7,8,9]. The sensitive early red cells are produced over a very limited time period so that a single exposure to the drug can result in a high proportion of cell deaths [5]. If sufficiently severe the embryos died, but in surviving embryos malformations were induced. Limited data in primates suggest that artemisinins may have a similar mechanism of action in the monkey leading to anaemia and embryolethality. However, the monkeys required more than 12 days of treatment to induce such embryonic death. No malformations were observed in the primate studies but these were limited in scope [1,5]. The significant difference between rodents and monkeys indicated that the sensitive period of artemisinins could be a longer time period in humans because in comparison with human may hold true from non–human primates than from rodents.

Furthermore, the preclinical studies of the reproductive toxicity in animal species (mice, rats, hamsters, guinea pig, rabbits and monkeys) indicated an important result that the injectable AS has the greatest toxicity to animal embryos. Following intravenous, intramuscular, and subcutaneous administrations, AS showed a less than 1.0 mg/kg of 50% fetus resorption dose (FRD_50_), which is much lower than the therapeutic dose of 2–4 mg/kg in humans. However, those animal species with oral artemisinins and intramuscular artemether (AM) were shown much safer than injectable AS in such an evaluation with 6.1–51.0 mg/kg of the FRD_50_ (Table 1). The mechanism of developmental severe embryotoxicity in animals after injectable AS was not known in 2006. It was not clear whether the toxicokinetic (TK) profiles were major role to produce the severe embryotoxicity after injectable AS.

More recently, primary antiangiogenic, anti–vasculogenic and antierythropoietic effects of dihydroartemisinin (DHA), an active metabolite of AS, *In vivo* and *in vitro* has been demonstrated in the embryos of rats and frogs [10,11,12,13,14,15]. The embryotoxicity, toxicokinetics, and tissue distribution of intravenous and intramuscular AS in pregnant and non–pregnant animals have been also conducted [16,17]. These data demonstrated that the severe embryotoxicity induced by injectable AS is because (1) injectable AS can provide much higher peak concentration than oral artemisinins and intramuscular AM; (2) DHA plays a key role in the embryotoxicity; (3) the highest conversion rate of AS to DHA among all artemisinins; (4) the conversion rate of AS to DHA was significantly increased in the pregnant animals; (5) the buildup of high peak concentrations of AS and DHA totally in the blood of pregnant rats was also significantly higher than those of the non–pregnant animals; and (6) the injectable AS can also make available higher distribution of AS and DHA in feto–placental tissues in the pregnant animals [16].

The purpose of this review is to evaluate and discuss the new reports and data recently achieved on the embryotoxic mechanism of artemisinins in rodents and monkeys. There are concerns in the difference on the drug sensitive period between rodents and primates, the difference on pharmacokinetic profiles following various administrative routes of artemisinins, and the difference on the embryotoxicity between pregnant animal species and women. The perspectives for pregnant women safety in artemisinins therapy will cover that progress and concerns to the assessment approaches which drug sensitive period can be clearly evidenced for rodents only, which current pharmacokinetic profiles can be inducing severe embryotoxicity, and, which risk in the reprotoxicity of artemisinins for the pregnant women may be lacked or avoided.

## Drug Sensitive Period in Rodent, Monkey, and Human Embryos

Studies in rodents have demonstrated that artemisinins can induce fetal death and congenital malformations at the low dose levels, which can be induced in rodents only within a narrow window in the early embryogenesis. The fetus and neonate are very sensitive and delicate. Events occurring during this period can, therefore, have a very significant influence on later life. The sensitive window for developmental embryotoxicity of artemisinins (DHA, AS, AM and arteether (AE)) in the rodents was identified as gestation days (GD) 10 to 14 [16,17,18]. The developmental toxicity has been observed in rats following treatment on single days between GD 10 and 14 when AS was administered orally at 17 mg/kg [15]. GD 11 was the most sensitive day for the induction of embryolethality, and GD 10 was the most sensitive day for the induction of malformations (cardiovascular defects and shortened and/or bent long bones). No developmental toxicity was seen following administration of the same dose administered on GD 9 or following 30 mg/kg on GD 16 or 17. The *In vivo* studies showed that four artemisinins: DHA, AS, AM and AE, administered orally to pregnant rats on GD 10 caused nearly equivalent effects in terms of embryolethality and teratogenicity (cardiovascular defects and shortened and/or bent long bones). This suggests that embryotoxicity (lethality and teratogenicity) is an artemisinin class effect [15,16]. 

A recent study in cynomolgus monkeys found that 12 mg/kg/day and 30 mg/kg/day AS treatment given on GD 20 to 50 caused embryo death between GD 30 and 40. The no–adverse–effect-level was 4 mg/kg/day. No malformations were observed in four surviving fetuses in the 12 mg/kg/day group but the sample size is not adequate to conclude that artesunate is not teratogenic at that dose in monkeys. All three live embryos in the 30 mg/kg/day artesunate group dosed from GD 20 and removed by caesarean section on GD 26, 32 and 36 respectively had marked reductions in erythroblasts. Treatments on GD 29 to 31 (3–day treatment) and GD27 to 33 (7–day treatment) did not cause embryolethality or changes in bone lengths at 12 mg/kg/day. The dose caused marked embryolethality when administered throughout organogenesis (GD 20–50). Since embryo death was observed only after more than 12 days of treatment at daily 12 mg/kg. The lack of developmental toxicity at this dose and treatment duration indicates that a shorter treatment period decreases the potential for AS–induced embryotoxicity in the monkeys [19].

Primitive erythroblasts develop in the visceral yolk sac and are released into the embryonic circulation on GD 10 in the rats, at about the same time that the heart begins to beat. If the primitive erythroblasts are also the primary target of AS action in the monkey, then the most sensitive window would be when those cells predominate in the embryonic circulation. In the cynomolgus monkey, the heart starts beating at about GD 18. Although no data to proof the timing of the switchover from primitive to definitive erythroblasts in monkeys at this time, the erythroblasts visible in the sections of embryos from GD 26, 32, and 36 were > 90% of blood cells were nucleated, suggesting that they were probably primitive erythroblasts during GD 18 to 36. On GD 50, only 9% of blood cells were nucleated, indicating that the transition from primitive to definitive erythroblasts was nearly complete on GD 50 [19]. The fact that a 3–day or 7–day treatment period at 12 mg/kg/day did not cause AS–related embryo deaths indicates that either the treatment period was too short to produce a depletion of embryonic erythroblasts or that the primate embryo can overcome embryonic erythroblast depletion for short periods of time.

The time window of sensitivity observed in the animal studies would correspond in the human to part of the first trimester (from conception through week 13) during organogenesis. Currently available information is inadequate for define precisely the likely period of maximum sensitivity in humans. Yolk sac haematopoiesis extends from GD 14 to week 6 in humans, and the onset of circulation begins at approximately GD 21 to 23. In a woman, the mesoderm of the yolk sac has been shown to exhibit localized thickenings, probably representing the primordial blood islands, at around GD 16. The blood islands are composed of hemoangioblasts–precursors of primitive erythroblast and endothelial lineages. The earliest primitive erythrocytes are formed in the yolk sac from GD 18.5 [19,20]. The onset of blood circulation coincides with the onset of the embryonic heartbeat, which probably occurs between GD 19 and GD 21 in humans, evidenced by the appearance of primitive erythrocytes in the cardiac cavity. The liver is the first organ to be colonized by the yolk sac and is the main site of definitive erythropoiesis around 5 through 24 weeks of gestation [21]. Primitive erythrocytes are the predominant circulating form in the first 8 to 10 weeks of gestation. Liver–derived definitive erythrocytes begin to enter the circulation by 8 weeks of gestation, but do not predominate until 11 to 12 weeks [22]. All available studies agree that yolk sac haematopoiesis disappears completely after the GD 60 [20]. 

In conclusion, the timing of the switchover from primitive to definitive erythroblasts is GD10–14 for rats, GD 18–50 for monkeys, and GD 16–60 for humans. Based on this information, if human embryos were sensitive to AS or DHA in the same way as rat and monkey embryos, then the most sensitive period for development toxicity induced by artemisinins would be predicted to begin with the onset of circulation in week 4 of gestation and end at approximately week 9 to 10 of gestation in humans. This means that the nucleated primitive erythroblasts have been largely replaced by non–nucleated definitive erythroblasts [19]. If primitive erythrocytes are formed over a longer period than that in rodents, then (unlike rats) much more daily doses (>12 days) may be required to produce a severe effect on the early blood cell population in primates and humans [5,19]. 

Although no animal species exists with which the situation in man can be completely mimicked, above comparison with human may hold true from non–human primates. With animal experiments only certain aspects of the whole complex situation can be analyzed. In order to achieve this successfully, animal species and experimental set–up have to be chosen carefully to represent the situation existing in humans in as suitable a model as possible. The more the model deviates from the situation existing in humans, the less will be the predictability. Today more information is available on the pharmacokinetic and toxicokinetic properties of artemisinins. This will supply data on the embryotoxic/teratogenic doses of a substance or on their non–embryotoxic/teratogenic doses relevant to man. In addition, the relative duration of exposure to three day ACT for malaria in humans, with respect to the duration of organogenesis, may be too short to induce the severe embryotoxicity. Further work is necessary to elucidate this aspect of embryogenesis in humans.

## Pharmacokinetic Profiles of Oral and Injectable Artemisinins

There are physiological changes in pregnancy that can cause a decrease in plasma drug concentrations and area under curves (AUCs), resulting in reduced efficacy [23]. This is likely due to increased clearance, larger volume of distribution and perhaps altered absorption following the oral administration. Clearly, oral dosages of these antimalarials need to be adapted to keep efficacy when given to pregnant patients and animals with malaria [24]. However, the pharmacokinetic parameters for the antimalarial agents show no significant difference between pregnant and non–pregnant women and animal species after single intravenous or intramuscular injections [16,25,26,27]. 

Preclinical studies of the reproductive toxicity in pregnant rats indicated an important result that the injectable AS has the severe toxicity to animal embryos by routes of intravenous, intramuscular, and subcutaneous administrations with the dose at <1.0 mg/kg. However, animals with oral artemisinins and intramuscular AM were shown much safer than injectable AS with the dose at 6.1–51.0 mg/kg (Table 1). Similar finding showed that single dose of 17 mg/kg oral AS and 1.5 mg/kg intravenous AS administered on GD 11 both caused 100% embryolethality and are close to the threshold for that effect (10 mg/kg oral AS caused only 15% resorptions and 0.75 mg/kg intravenous AS caused 7% resorptions) [18,28]. Toxicokinetic and tissue distribution data demonstrated that the severe embryotoxicity induced by injectable AS is because of following six factors [16].

- Injectable AS can provide much higher peak concentration (3–25 folds) than oral artemisinins and intramuscular AM in animals [16]. *In vitro* results evidently indicated that the drug exposure level and time are important to induce the embryotoxicity [11,12,13]. However, *In vivo* studies the drug exposure level is more important than drug exposure time because AS and DHA had very short half–lives (< 1 h) in animal species [16].
- AS is completely converted to DHA and is basically a prodrug of DHA. Also, DHA was more effective than AS in inhibition of angiogenesis and vasculogenesis *in vitro* [11,12,13,14,15]. In addition, without DHA formation, the embryotoxicity of artemisinins can be reduced by using artemisone, which is significantly less anti–angiogenic activity than DHA [10,29], which is a novel derivative of artemisinin and is not metabolized to DHA. However, at the same doses, artemisone does not inhibit of angiogenesis. Therefore, DHA seems to play an essential role in the embryotoxicity;
- The highest conversion activity of AS to DHA is shown among all artemisinins. The conversion rate of AS to DHA was 38.2–72.7% in comparison to that of AM and AE rate of 12.4–14.2% [16].
- Difference to single dose, the conversion rate of AS to DHA was significantly increased in the pregnant animals than that in the non–pregnant rats following multiple injections. The concentrations of DHA generated in pregnant rats were 2.2–fold higher on day 1 and 4.5–fold higher on day 3 than that in the non–pregnant animals, resulting in a total AUC_D1–3_ (15,049 ng·h/mL) that were about 3.7–fold higher in pregnant rats than that (4,015 ng·h/mL) in non–pregnant rats during daily for three days of treatment. The ratios of AUC_DHA_/AUC_AS_ were also shown 0.99–1.02 for pregnant rats and 0.42–0.48 for non–pregnant animals, indicating that the total exposure of pregnant rats to DHA during the whole period of treatment was much higher than that in non–pregnant rats [16].
- The buildup of high peak concentrations of AS and DHA totally in the plasma of pregnant rats was significantly higher than those of the non–pregnant animals after repeated dosing. In comparison to the toxicokinetics of AS revealed that the peak concentration (C_max_) of AS (16,545–14,927 ng/mL) in pregnant rats was double higher than that (8,668–5,037 ng/mL) in non–pregnant animals. The plasma concentration of AS increased from AUC 3,749 ng·h/mL on day 1 to 4,758 ng·h/mL on day 3 in the pregnant rats, but on the contrary the AS decreased from AUC 3,984 ng·h/mL to 2,239 ng·h/mL from day 1 to day 3 in the non–pregnant animals. Similarly, the mean peak concentration (C_max_) of DHA, an active metabolite of AS, (10,335–9,087 ng/mL) in pregnant rats was more than three times higher than that (3,049–2,409 ng/mL) in non–pregnant animals from day 1 to day 3. Comparable to C_max_ values, the mean AUC data of DHA were also much higher in pregnant animals (3,681–4,821 ng·h/mL) than that in non–pregnant rats (1,636–1,049 ng·h/mL). The TK results were also exhibited the mean AUC of DHA were significantly increased from day 1 (3,681 ng·h/mL) to 3 (4,821 ng·h/mL) in the pregnant rats, but remarkably decreased from the day 1 (1,636 ng·h/mL) to the day 3 (1,049 ng·h/mL) in the non–pregnant animals [16].
- The injectable AS can also make available higher distribution of AS and DHA in the tissues of feto–placental units in the pregnant animals after the multiple administrations. The tissue distribution study of ^14^C–AS showed that the total AUC_0–192h_ of the radioactivity was 22,879 µg equivalents·h/g in all measured tissue of the pregnant rats. The 6.54% (1480 µg equivalents∙h/g) of the total radioactivity was present in all the feto–placental tissues. During the 192 h treatment period, measured levels of radioactivity in the ovary, placenta, and uterus was 555, 367 and 216 µg equivalents·h/g, respectively. This was more than 2–4 folds higher than in blood with 134 µg equivalents∙h/mL. Tissue/blood partition coefficients (*K*_t:b_) of radiolabeled AS are highly observed in placenta (2.75), uterus (1.61) and ovary (4.16). TK data also showed that AS and DHA concentrations in the blood of the pregnant rats were significantly higher (1.5 to 3.7-fold) than those of the non–pregnant animals. The half–life of radioactivity was measured in the blood at 97.73 hr, whereas that in the ovary, placenta, and uterus were 160, 201, and 153 hr, respectively, suggesting that ^14^C–AS remained in those reproductive tissues longer than in blood [16].

Conventionally, the pharmacokinetics of antimalarials is altered in pregnancy after oral administration and the drug plasma level is decreased. However, above researches showed that AS and DHA concentrations in the plasma and reproductive tissues of pregnant rats were significantly increased than that in the non–pregnant animals after injectable AS [16]. The significantly increase of AS and DHA concentration in animals may highly relate to the severe embryotoxicity of injectable AS even at a low–dosage regimen in the pregnant animals. 

## Possibly Safe with Oral Artemisinins in Pregnant Women

There are three issues demonstrated that the oral artemisinins may be safe in the pregnant women: (1) the pharmacokinetics of antimalarials is altered in pregnancy after oral administration, which can cause a decrease of drug exposure level; (2) the oral artemisinins produced only low peak concentration compared to injectable artemisinins as discussed above; and (3) data on clinical trials regarding the possible effects of artemisinins on pregnancy have not shown any embryotoxic effects in humans for the past 20 years with oral artemisinin monotherapy or oral ACTs. 

The physiological changes in pregnancy–beginning during the first trimester, and most marked during the third trimester–alter the absorption, distribution and clearance of drugs. In addition, most drugs gain access to the feto–placental unit. The pharmacokinetics of antimalarials is altered in pregnancy after oral administration. This is the consequence of multiple factors: expansion of the distribution volume, increase in clearance, change in the protein binding, lipid distribution and absorption of drugs, as well as an influence of hormonal changes on the drug metabolism [23]. These physiological changes in pregnancy can cause a decrease of drug exposure levels, resulting in reduced efficacy.

Few studies in women have been published for artemisinins and other antimalarials. For example, chloroquine in oral treatment [30] by using prophylaxis [31], oral mefoloquine [32], oral progunil [33,34], oral atovaquone [35], as well as oral DHA [36] all have altered kinetics in pregnancy, and plsama levels are significantly lower than in non–pregnant patients with malaria [30,31,32,33,34,35,36]. This is likely due to increased clearance, larger volume of distribution and perhaps altered absorption following the oral administration. In comparison to non–pregnant Thai women, C_max_ and AUC of DHA values were 4.2 and 1.8 times lower in pregnant Karen patients [26,36,37]. A similar observation is also found in animal studies for oral administration of AS [38]. Clearly, the oral dosages of these antimalarials need to be adapted to keep efficacy when given to pregnant patients and animals with malaria [24]. In this case, the oral drugs appear safe due to the less drug exposure and fast elimination in the pregnancy. 

The adverse impact of malaria in pregnant women is largely caused by *P. falciparum*, and approximately 95% of clinical cases globally occur in Asia and sub–Saharan Africa. Every year there are approximately 50 million pregnancies in women living in malarious areas [27]. Artemisinins have been used to treat pregnant women since 1989 (Table 2). 

There is now a reasonable body of evidence for safety from the most of the clinical trials published from 1989 to 2009 in nearly 1,837 pregnant women exposed to an artemisinin agent or ACT with 176 pregnant patients in the first trimester. There were no clinically significant adverse effects of the drug, neither in the outcomes of the pregnancies, nor in the development (neurological and physical) of the infants, including 44 infants exposed during the first trimester [2,3,39,40,41,42,43,44,45,46,47,48,49,50,51,52,53,54,55,56,57]. Recent data published by WHO [5] presented the evidence on artemisinin exposure in pregnancy from ongoing studies in Thailand, Zambia and Bangladesh. Data will be published fully in due course. In Thailand, 1,530 first trimester exposures to a range of antimalarial medicines include 170 pregnant women treated with artemisinins. Irrespective of the antimalarial medicine used, the higher the number of episodes of *P. falciparum* and the greater the number of times the women had to be treated in the first trimester, thegreater the chance of abortion. In addition, fever, hyperparasitemia and older maternal age were significant positive risk factors for an abortion in the first trimester, whereas antimalarial drug treatments were not significantly related.

To evaluate the data showed that more than 917 pregnant women (including 123 in the first trimester) have received a monotherapy of artemisinins, and more than 920 pregnant patients (including 53 in the first trimester) have received an ACT with no increase in adverse outcomes (Table 2). It was concluded that there is insufficient evidence at present to warrant a change in current WHO policy recommendations on the use of ACTs for the treatment of malaria in pregnancy. Current WHO Guidelines recommend that in uncomplicated malaria, ACT should be used in the second and third trimester, but should be used in the first trimester only if it is the only effective treatment available [1]. Consequently, these are still valid. However, the medicine of choice for initial treatment in the first trimester of pregnancy varies because of differences in drug sensitivities in different regions. At least, the immediate use of artemisinins is justified in situations where the first treatment fails because of the dangers of repeated malaria infections in pregnancy. Furthermore, the ACTs may be used to treat pregnant women in all trimesters after further safety studies based on the above issues discussed.

## Other Possible Considerations and Further Studies

Are artemisinins really not toxic to either the women, the fetuses during pregnancy, or to the infants during lactation? Without other relevant pharmacokinetic data, drug sensitive period and embryotoxicity studies in humans, it is difficult to quantify and predicate the risk of possible embryonic death or teratogenicity with exposure to artemisinin compounds in the first trimester in women. However, the three factors discussed above: 1) the drug sensitive period in human and animal species, 2) the pharmacokinetic characteristics (including tissue distribution), and 3) a strong safe evidence from clinical trials may assist us in avoiding the reprotoxicity (low birth weight, abortion, and even potentially fetal death) for pregnant women requiring malaria therapy.

In accordance with WHO recommendations and the new researches described above, the two major issues for considering artemisinin drug use in a program for prevention or management of malaria in pregnant women are safety and effectiveness [1]. First, the exposure to the injectable AS should be cautious, during the early sensitive days (GD 15 to week 6 in humans), which is the likely critical period for induction of embryo damage. This is essentially the same recommendation as WHO consult above that the artemisinin drugs should not be used in the first trimester of pregnancy in women. Secondly, in uncomplicated malaria WHO recommends that the oral ACTs should only be used in the second and third trimester when other treatments are considered unsuited. However, we feel that oral regimens could be used to treat pregnant women at in all trimesters when other treatments are considered unavailable, because the common oral regimens provide a lower peak concentration and short exposure time, and that can make the agents safer than intravenous or intramuscular injection of AS on the embryotoxicity. 

In severe malaria, WHO recommends that artemisinins are preferred over quinine in the second and third trimester because of the hypoglycemia associated with quinine. However, in the first trimester until more evidence becomes available on the risk benefit ratio of artemisinins, both artesunate and quinine may be considered as options. In severe malaria treatment should be started without delay and whichever medicine is immediately available should be used [1]. If it is possible, reliable pharmacovigilance on the use of these drugs in pregnancy and the careful monitoring of safety after exposure in the first trimester of pregnancy, when treatment may occur inadvertently or be necessary to save life even with injection AS, are needed. 

It remains unknown how humans have been able to avert the death embryotoxicity described in animal species. Therefore, further studies are needed to define the precise mechanism of damage in animal models are warranted [5]. (1) There is a need to understand more fully the critical period of exposure, and the duration of exposure necessary to induce embryotoxicity in primates. (2) There is a need to perform embryotoxicity studies on newer artemisinins (synthetic trioxanes and modified artemisinins) and other peroxidic molecules in order to evaluate their potential for developmental toxicity. (3) There is a need for metabolic profile studies in rodents and primates to compare their profiles of metabolism with that in humans. (4) Whole embryo culture studies *in vitro* should be extended to investigate the role of metabolites, oxygen and reactive species. Studies on the toxic activity of rat and human blood following artemisinin administration would give an indication of the presence of active metabolites. 

More studies are needed to define the clinical safety of artemisinins or ACTs in pregnant women are necessitated [5]. (1) It is not known whether human’s primitive erythroblasts occur similarly to that observed in rodents, leading to a period of heightened sensitivity to artemisinins. Further work is necessary to elucidate this aspect of embryogenesis in humans. (2) There is a need for a review of the safety of all antimalarial medicines in pregnancy, especially when used during the first trimester. In particular an up–to–date review of pregnancy outcomes following exposure to artemisinins during the first trimester is required urgently. (3) Since 2002, population studies have been carried out by WHO and others in Bangladesh, Kenya, South Africa, the United Republic of Tanzania and Zambia. Some of these studies have included the drug exposures of pregnant women to artemisinins. These data should be made available for review by experts in reproductive epidemiology with a view to assessing the strength of the information involving first–trimester exposures. (4) There is a need to establish how we can move ahead to obtain the required information about safety in pregnancy.

## Conclusions

Infection with *Plasmodium falciparum* malaria in pregnancy is dangerous to both mother and her child, so efficacious and safe treatment is important. In animal work, there is clear evidence of death of embryos and some evidence of morphological abnormalities in mice, rats, hamsters, guinea pig, rabbits and monkeys in early pregnancy by using artemisinins [16]. The mechanisms and the pharmacokinetic profiles that affect reproductive toxicity in animal species are currently understood. However, it remains unknown how these findings translate to man [5,39]. Data from limited clinical trials in pregnant women (1,837 cases) exposed to artemisinin compounds and ACTs, including a small number (176 cases) in the first trimester, have not shown an increase in the rates of abortion or stillbirth; they have also not shown evidence of abnormalities. With regard to acute toxicity, humans appear to be less sensitive than animals [58,59] and humans have much better repair capabilities than do animals [60]. Other possible considerations for the reprotoxicity discrepancy observed between animal and human:

- In the rodent, the sensitive early red cells are over a very limited time period can result in a high proportion of cell deaths [1]. In contrast, primates required a longer period of treatment of 12 days to induce such embryonic death [19]. In humans only limited information is available about this stage of red cell development; however, it is known to take place over a longer time period, and it may well be in treatment of two to three days for malaria would not produce serious toxic effects in humans.
- The animal data revealed that only injectable AS (intramuscular, intravenous, or subcutaneous) induces reprotoxicity at the lower dose (0.6–1.0 mg/kg) than the therapeutic dose (2–4 mg/kg) in humans. Other doses in different regimens (oral artemisinins or intramuscular AM) are safe at the higher levels (6.1–51.0 mg/kg) than the therapeutic doses. Current oral intake, the most commonly used route of administration in pregnant women with ACTs, results in lower peak concentration and shorter exposure, which concentration–course is unlikely to induce the embryotoxicity. Since more than 99% of pregnant patients have been treated with oral artemisinins or intramuscular AM in our counted trials (1,837 cases), it may be the reason for the lack of toxicity observed.
- Many of the pregnant patients followed have been seriously ill with malaria, which is responsible for 5–12% low birth weight (LBW), 35% of LBW that is preventable during pregnancy [61], and contributes to 70,000–200,000 infant deaths each year [62]. The resulting evidence may lead to future studies on whether the pattern of reprotoxicity has occurred from the illness or from the drug(s) [63].
- A number of early reports have not paid much attention to the feasibility of associating low birth weight, abortion, and/or infant disorders in patients with such treatment of malaria with artemisinins. None of these studies had adequate power to rule out rare serious adverse events, even in second and third trimesters and there is not enough evidence to effectively assess the risk–benefit profile of artemisinins for pregnant women, particularly for first trimester exposure.
- Post–marketing surveillance has been limited in developing countries, and the potential reproductive side–effects of the drug have not been well recorded.

The reproductive toxicity of oral artemisinins and intramuscular AM is not possibly happened in humans with current knowledge in the embryotoxic mechanism and pharmacokinetic researches in current dose regimens. The possible embryotoxicity should be avoided in lock of the exposure of artemisinins in these sensitive days (humans in first trimester), which the critical period for induction of embryo damage and resorption. In addition, to protect pregnant women from the embryotoxicity with treatment of artemisinin derivatives, the injectable AS should be in cautious use. There is agreement that the artemisinin derivatives should not be withheld at any stage of pregnancy, in cases of severe and complicated malaria, if the life of the mother is at risk. It is considerable that the oral regimens of artemisinins are much safer than parenteral administrations in pregnant patients. When relating the animal and human toxicity of artemisinins, the different sensitive period and pharmacokinetic profiles may possibly provide a great margin of safety in the pregnant women.

## Figures and Tables

**Table 1:** Embryotoxic effects (NOAEL and FRD_50 or 100_) of artemisinin (QHS), dihydroartemisinin (DHA), artesunate (AS), artemether (AM) and arteether (AE), given intragastrically (Oral), intravenously (IV), intramuscularly (IM), and subcutaneously (SC) in pregnant mice, rats, hamster, guinea pig, rabbits, and monkeys [16,17,28].

Animal (drugs)	Dose duration	Dose regimens (daily)	Dosing route	No–observed–adverse–effect–level (NOAEL) on fetus resorption (mg/kg)				FRD_100_(mg/kg)	FRD_50_ (95% CL)(mg/kg)
				Oral	IM	IV	SC		
Mice (AM) (DHA) Rats (QHS) (QHS) (DHA) (AM) (AS) (AS) (DHA) (AM) (AE) (AS) (AS) (AM) (AS) (AS) Hamster (DHA) (DHA) (AS) Guinea Pig (DHA) Rabbits (AM) (AS) (DHA) Monkey (AS)	GD 6–15 GD 7 GD 1–6 GD 7–13 GD 9.5, 10.5 GD 6–15 GD 6–17 GD 10 GD 10 GD10 GD 10 GD 11 GD 6–15 GD 6–15 GD 11 GD 6–13 GD 7 GD 7 GD 5 GD 18 GD 7–18 GD 7–19 GD 9 GD 20–50	Multiple x 10 Single Multiple x 6 Multiple x 7 Single Multiple x 10 Multiple x 12 Single Single Single Single Single Multiple x 10 Multiple x 10 Single Multiple x 13 Single Single Single Single Multiple x 12 Multiple x 13 Single Multiple x 12	IM SC Oral Oral Oral Oral Oral Oral Oral Oral Oral Oral SC IM IV IV, IM SC Oral SC IM IM Oral IM Oral	5.6 7 7.5 2.5 5–7 20 5–7 4	5.4 2.7 0.5 2.5 0.7 5.0	0.75 0.4	10 0.2 4.2 0.35	15.0 11.1 19.4 20.3 17.0 1.5	11.3 (10.6 – 12.0) 32.8 (27.7 – 38.9) 11.5 (10.5 – 12.2) ≥ 35 NA 14.4 (10.4 – 17.8) 7.74 (6.92 – 8.57) NA NA NA NA NA 0.58 (0.55 – 0.61)* 6.13 (4.52 – 8.26) NA 0.60 – 0.61 (0.47–0.72)* 6.06 (5.90 – 6.21) 51.0 (37.9 – 68.7) 1.0 (0.9 – 1.2)* 18.3 (13.9 – 24.2) NA NA 7.57 (7.48 – 7.67) ≥ 12

FRD_50_ or _100_ = drug dose induces 50% or 100% fetus re–absorbed; GD = gestation day (The day of mating was defined as day 0 of gestation.); * The severe toxic effects were detected in the animals treated with AS after single or multiple intramuscular, intravenous or subcutaneous injections. Values of ED_50_ are given as median (95% confidence limits). NA = not available.

**Table 2:** Clinical trials and safety evaluation of oral and injectable artemisinins following monotherapy and artemisinin–based combination therapy (ACT) in pregnant women from 1989 to 2009 [39,40,41,42,43,44,45,46,47,48,49,50,51,52,53,54,55,56,57,58].

Author (Year)	Drugs	Regimen	No of patients	Trimester (Patients)	Clinical observation in safety	Refs
**Monotherapy** Wang (1989) Guo (1990) Li (1994) Sowunmi (1998) McGready (1998) McGready (2001) McGready (2001) Adam (2004) McGready (2008) Adam (2009) **ACTs** McGready (2000) Bounyasong (2001) Deen (2001) McGready (2003) McGready (2005) Adam (2006) Kalilani (2007) McGready (2008) Kaye (2008) Rijken (2008) Mutabingwa (2009)	QHS, AM QHS, AS, AM QHS, AS, AM AM AS, AM AS AS, AM AM AS AM AS+MQ AS+MQ AS+SP AS+AP AS+AP AS+SP AS+SP AL AL DHA–PPQ AS+AQ	Oral, IM Oral, IV, IM Oral, IV, IM Oral Oral, IM Oral Oral, IV IM Oral IM, Oral Oral Oral Oral Oral Oral Oral Oral Oral Oral Oral Oral	6 6, 2, 11 21 45 83 64 459, 2 28 128 48, 14 66 60 287 27 39 32 47 125 110 50 77	2 2, 3 2 2, 3 1 (16), 2, 3 (67) 2, 3 1 (44), 2,3 (417) 1 (1), 2,3 (27) 2, 3 1 2, 3 2, 3 1 (50), 2, 3 (237) 1 (3), 2, 3 (24) 2, 3 2, 3 2, 3 2, 3 2, 3 2, 3 2, 3	none found evidence of drug toxicity no premature delivery or death–in–utero reported No side–effects do not produce undue deleterious effects Caution: 20% abortions in first trimester group. well–tolerated and safe for the mother and the fetus well tolerated with no evidence of adverse effects well tolerated with symptom–free within three days well tolerated and safe. may be safe to use during early pregnancy (2 miscarriages) AS–MQ was significantly better tolerated ACT might be the alternative treatment in pregnancy.. well–tolerated and safe for the mother and the fetus. well tolerated and no toxicity for the mothers and fetus. AS–AP is a well–tolerated, and safe for the mother and the fetus. the drug was well tolerated, with symptom–free within 2 days. Safe, well tolerated and side–effects during pregnancy. Well tolerated and safe. safe to use in treatment of uncomplicated malaria in pregnancy. well tolerated and no toxicity for the mothers or the fetus. the combination tested was efficacious and appeared safe.	[39] [40] [41] [42] [43] [44] [2] [45] [46] [47] [48] [49] [50] [51] [52] [3] [53] [46] [54] [55] [56]

QHS = Artemisinin; AM = Artemether; AS = Artesunate; AS + MQ = Artesunate + Mefloquine; AS + SP = Artesunate + Sulfadoxine–Pyrimethamine; AS + AP = artesunate + Atovaquone–Proguanil; AL = Artemether–Lumefantrine; AS + AQ = Artesunate + Amodiaquine; DHA–PPQ = Dihydroartemisinin–Piperaquine. IM = intramuscular; IV = intravenous; Refs. = References.
